# 

**Published:** 1883-10

**Authors:** 


### CONTENTS.

### COMMUNICATIONS.

Dental Nomenclature........................................................................ 451
Anæsthetics.................................................................................. 467

### SELECTIONS.

The Disinfecting Power of Antiseptics........................................... 487
Testing the Purity of the Atmosphere.............................................. 489
Successful Exsection of Inferior Dental Nerve for Obstinate Neuralgia; Bone Grafting.............................................................. 491
The Effect of Tobacco on Youth...................................................... 492
Half-Breed Micro-Organisms ........................................................... 493
Note Upon a Method of Root Filling................................................. 498
Case of Antral Abscess of Twenty Years' Standing—Extraction of Root of Right Lateral Incisor—Recovery.................................. 499
Inheritance as a Cause of Drunkenness........................................... 501
Unjustifiable Use of Chloroform...................................................... 501
Treatment of Tumors by Electrolysis.............................................. 502
Effect of Alum Gargles Upon the Teeth........................................... 502
Insufficiency of Food in Certain Cases........................................... 503
Instrumental Cases......................................................................... 504
The Poisons in Tobacco Smoke....................................................... 504
Nitrous Oxide and Chloroform as an Anesthetic.............................. 505
The Effect of Alcohol on the Fœtus Through the Blood of the Mother 506
Milignant Pustule Communicated by a Fly ..................................... 507
The Medical Voyage of Life ............................................................ 508
Human Obesity ............................................................................... 508

### EDITORIAL.

Journalism ...................................................................................... 509
A Case—Amalgam ........................................................................... 510
Ohio State Dental Society................................................................ 511
State Board of Dental Examiners.................................................... 512

